# Fast parallelized sampling of Bayesian regression models for whole-genome prediction

**DOI:** 10.1186/s12711-020-00533-x

**Published:** 2020-03-23

**Authors:** Tianjing Zhao, Rohan Fernando, Dorian Garrick, Hao Cheng

**Affiliations:** 1 Department of Animal Science, University of California Davis, Davis, CA 95616 USA; 2 Integrative Genetics and Genomics Graduate Group, University of California Davis, Davis, CA 95616 USA; 3 Department of Animal Science, Iowa State University, Ames, IA 50011 USA; 4 School of Agriculture, Massey University, Ruakura Research Centre, Hamilton, New Zealand

## Abstract

**Background:**

Bayesian regression models are widely used in genomic prediction, where the effects of all markers are estimated simultaneously by combining the information from the phenotypic data with priors for the marker effects and other parameters such as variance components or membership probabilities. Inferences from most Bayesian regression models are based on Markov chain Monte Carlo methods, where statistics are computed from a Markov chain constructed to have a stationary distribution that is equal to the posterior distribution of the unknown parameters. In practice, chains of tens of thousands steps are typically used in whole-genome Bayesian analyses, which is computationally intensive.

**Methods:**

In this paper, we propose a fast parallelized algorithm for Bayesian regression models called independent intensive Bayesian regression models (BayesXII, “X” stands for Bayesian alphabet methods and “II” stands for “parallel”) and show how the sampling of each marker effect can be made independent of samples for other marker effects within each step of the chain. This is done by augmenting the marker covariate matrix by adding *p* (the number of markers) new rows such that columns of the augmented marker covariate matrix are orthogonal. Ideally, the computations at each step of the MCMC chain can be accelerated by *k* times, where *k* is the number of computer processors, up to *p* times, where *p* is the number of markers.

**Results:**

We demonstrate the BayesXII algorithm using the prior for BayesC$\pi$, a Bayesian variable selection regression method, which is applied to simulated data with 50,000 individuals and a medium-density marker panel ($\sim$ 50,000 markers). To reach about the same accuracy as the conventional samplers for BayesC$\pi$ required less than 30 min using the BayesXII algorithm on 24 nodes (computer used as a server) with 24 cores on each node. In this case, the BayesXII algorithm required one tenth of the computation time of conventional samplers for BayesC$\pi$. Addressing the heavy computational burden associated with Bayesian methods by parallel computing will lead to greater use of these methods.

## Background

Genome-wide single nucleotide polymorphism (SNP) data have been adopted for whole-genome analyses, including genomic prediction [1] and genome-wide association studies [2]. Bayesian regression models are widely used in genomic prediction, where the effects of all markers are estimated simultaneously by combining the information from the phenotypic data and priors for the marker effects and other parameters such as variance components or membership probabilities. Most of the widely-used Bayesian regression models differ only in the prior used for the marker effects. For example, the prior for each marker effect in BayesA [1] follows a scaled t-distribution, whereas in variable selection regression, the prior for each marker effect is a mixture distribution, such as BayesB [1], BayesC [3], BayesC$\pi$ [4] and BayesR [5, 6].

In these Bayesian regression models, closed-form expressions for the marginal posterior distributions of parameters of interest, e.g., marker effects, are usually not available. Thus, inferences from most Bayesian methods are based on Markov chain Monte Carlo (MCMC) methods, where statistics are computed from a Markov chain that is constructed such that the stationary distribution of a random vector ${\mathbf {x}}$ is equal to the posterior. It has been shown that statistics computed from such a Markov chain converge to those from the stationary distribution as the chain length increases [7]. In Bayesian regression models for genomic prediction, the vector ${\mathbf {x}}$ has a length about equal to the number *p* of markers or a multiple of it if auxiliary variables such as locus-specific marker effect variances are introduced to the analysis as in BayesA or BayesB. A widely used method to construct such a Markov chain is Gibbs sampling. In Gibbs sampling, at step *t*, each component of the vector ${\mathbf {x}}_{t}$ is sampled from the conditional distribution of that component given all the other components sampled up to that point [8]. In some Gibbs samplers proposed for Bayesian variable selection methods such as BayesB [1, 9], BayesC [3] or BayesC$\pi$ [4], for example, within each step, each variable in the vector ${\mathbf {x}}$ is sampled conditionally on all the other variables. This includes, for each marker *i*, its effect, the effect variance and a Bernoulli variable indicating whether the effect is zero or non-zero, as well as all non-marker effects and the residual variance. These are examples of a single-site Gibbs sampler where each variable is iteratively sampled conditional on the current values of all other variables.

In practice, chains of about tens of thousands steps are typically used in whole-genome Bayesian regression models [10, 11], and within each step of the chain, Gibbs sampling requires iteratively sampling at least *p* unknowns. Medium-level marker panels are often used in whole-genome prediction projects in animal breeding, where *p* is about 50,000 or larger. Thus, Bayesian regression models are usually computationally intensive, and commonly used strategies to sample these *p* unknowns efficiently are demanding.

The Ergodic theorem of Markov chain theory states that statistics computed from an increasingly long chain converge to those from the stationary distribution [7], whereas no such theory demonstrates that convergence can be achieved from an increasing number of shorter chains. It has sometimes been suggested that parallel computing can be adopted in MCMC by using multiple processors to obtain several short chains in parallel and then pooling the statistics computed from these short chains. In this case, caution is needed to make sure these short chains have converged to the stationary distribution. Combining several short chains will reduce the Monte Carlo variance of the computed quantities, but this may not yield statistics from the stationary distribution. Thus, to rapidly construct a single long enough chain in parallel is key to addressing the problem of the long computing time in Bayesian regression models. This is difficult because a Markov chain is an iterative process, and it can not be broken into several independent processes.

Parallel computing has been used in whole-genome prediction with Bayesian regression models by parallelizing the computations, including vector additions and dot products, associated with each marker at each step of the chain. To parallelize the computations, vectors are split up and additions or products are done in parallel on multiple processors [12, 13]. These strategies can be used for most methods involving matrix or vector calculations. Speedup from these approaches, however, is limited, because marker effects still need to be sampled iteratively.

Another appealing approach is to parallelize the Gibbs sampling for all markers within each step of the chain. In a single-site Gibbs sampler, however, sampling of a marker effect is from the full conditional distribution, which is the conditional distribution of this marker effect given the current values of all the other markers. Thus, parallelizing Gibbs sampling would not be feasible unless the full conditional distributions do not depend on the values of the variables being conditioned on, i.e., unless the full-conditionals are independent. In this paper, we show how the full conditional distributions of the marker effects can be made independent within each step of the chain. This is done by augmenting the marker covariate matrix by adding *p* new rows such that columns of the augmented marker covariate matrix are mutually orthogonal. The phenotypes corresponding to the augmented rows of the marker covariate matrix are considered missing.

The objective of this paper is to propose a fast parallelized algorithm for Bayesian regression models that we call BayesXII, where “X” stands for Bayesian alphabet methods and “II” stands for “parallel”. In this paper, the prior for BayesC$\pi$, a Bayesian variable selection regression method, was used to demonstrate the BayesXII algorithm. Use of this approach with other priors, such as those in BayesA, BayesB or Bayesian Lasso, should be straightforward. Simulated data for 50,000 individuals with genotypes from a medium-level marker panel ($\sim$ 50,000 markers) were used to compare the computational efficiency of the BayesXII algorithm with the conventional sampler for BayesC$\pi$.

## Methods

### Bayesian linear regression models

In Bayesian linear regression models, for simplicity and without loss of generality, we assume that individuals have a general mean as the only fixed effect. Phenotypes of *n* genotyped individuals are then modeled as

$$ {\mathbf {y}}={\mathbf {1}}\mu +{\mathbf {X}}{\mathbf {a}}+{\mathbf {e}}, $$

where ${\mathbf {y}}$ is the vector of *n* phenotypes, $\mu$ is the overall mean, ${\mathbf {X}}$ is the $n\times p$ marker covariate matrix (coded as 0, 1, 2), ${\mathbf {a}}$ is a vector of *p* random additive marker effects and ${\mathbf {e}}$ is a vector of *n* random residuals. A flat prior is used for $\mu$. The prior for the residual ${\mathbf {e}}$ is ${\mathbf {e}}|\sigma _{e}^{2}\sim \ N(\mathbf 0,{{\mathbf {I}}\sigma }_{e}^{2})$ with $\left( \sigma _{e}^{2}\mid \nu _{e},S_{e}^{2}\right) \sim \nu _{e}S_{e}^{2}\chi _{\nu _{e}}^{-2}$. The columns of ${\mathbf {X}}$ are usually centered prior to further computation. In BayesC$\pi$, a Bayesian variable selection method, the prior for the marker effect is a mixture of a point mass at zero and a univariate normal distribution with null mean and a common locus variance $\sigma _{a}^{2}$ with $\left( \sigma _{a}^{2}\mid \nu _{a},S_{a}^{2}\right) \sim \nu _{a}S_{a}^{2}\chi _{\nu _{a}}^{-2}$ [3, 4].

#### Gibbs sampling for marker effects in Bayesian regression models

In Gibbs sampling, the full conditional distribution of $a_{j}$, the marker effect for locus *j*, when $a_{j}$ is non-zero, can be written as

$$ \left( a_{j}\mid ELSE\right) \sim N\left( \hat{a_{j}},\frac{\sigma _{e}^{2}}{{\mathbf {X}}_{j}^{T}{\mathbf {X}}_{j}+\frac{\sigma _{e}^{2}}{\sigma _{a}^{2}}}\right) , $$

where *ELSE* stands for all the other parameters and ${\mathbf {y}}$, ${\mathbf {X}}_{j}$ is the *j*th column of ${\mathbf {X}}$, and $\hat{a_{j}}$ is the solution to

1 $$ \left( {{\mathbf{X}}_{j}^{T} {\mathbf{X}}_{j}  + \frac{{\sigma _{e}^{2} }}{{\sigma _{a}^{2} }}} \right)\hat{a}_{j}  = {\mathbf{X}}_{j}^{T} \left( {\mathbf y - \mathbf 1\mu  - \sum \limits_{{j^{\prime} \ne j}} {{\mathbf{X}}_{{j^{\prime}}} a_{{j^{\prime}}} } } \right) $$

2 $$=  {\mathbf {X}}_{j}^{T}{\mathbf {y}}-{\mathbf {X}}_{j}^{T}{\mathbf {1}}\mu -\sum _{j^{\prime}\ne j}{\mathbf {X}}_{j}^{T}{\mathbf {X}}_{j^{'}}a_{j^{'}}. $$

The derivation of the full conditional distributions of other parameters of interest in Bayesian regression models are shown in Appendix.

### Parallelized Bayesian regression models (BayesXII)

In commonly-used Gibbs sampling, the sample for each marker effect, $a_{j}$, can not be obtained simultaneously, i.e., in parallel, because samples for other marker effects, $a_{j^{'}\ne j}$, appear in the term $\sum _{j^{'}\ne j}{\mathbf {X}}_{j}^{T}{\mathbf {X}}_{j^{'}}a_{j^{'}}$ on the right-hand-side of (2), i.e., the full conditional distributions of the marker effects are not independent. One solution is to orthogonalize columns of the marker covariate matrix ${\mathbf {X}}$ such that the term $\sum _{j^{'}\ne j}{\mathbf {X}}_{j}^{T}{\mathbf {X}}_{j^{'}}a_{j^{'}}$ in (2) becomes zero. A data augmentation approach [14] to obtain a design matrix with orthogonal columns is described below.

#### Orthogonal data augmentation (ODA)

Let $${\mathbf {W}}_{o}=\begin{bmatrix}{\mathbf {1}}&{\mathbf {X}}\end{bmatrix}$$ be the incidence matrix for the Bayesian regression analysis. Following Ghosh et al. [14], we show here how to augment ${\mathbf {W}}_{o}$ as $${\mathbf {W}}_{c}=\begin{bmatrix}{\mathbf {W}}_{o}\\ {\mathbf {W}}_{a} \end{bmatrix}$$ such that

$$ {\mathbf {W}}_{c}^{T}{\mathbf {W}}_{c}=\begin{bmatrix}{\mathbf {W}}_{o}^{T}&{\mathbf {W}}_{a}^{T}\end{bmatrix}\begin{bmatrix}{\mathbf {W}}_{o}\\ {\mathbf {W}}_{a} \end{bmatrix}={\mathbf {D}}, $$

where ${\mathbf {W}}_{a}$ is a square matrix of dimension $p+1$ and ${\mathbf {D}}$ is a diagonal matrix. Thus,

3 $$ {\mathbf {W}}_{a}^{T}{\mathbf {W}}_{a}={\mathbf {D}}-{\mathbf {W}}_{o}^{T}{\mathbf {W}}_{o}, $$

and ${\mathbf {W}}_{a}$ can be obtained using Cholesky decomposition (or Eigen decomposition) of, ${\mathbf {D}}-{\mathbf {W}}_{o}^{T}{\mathbf {W}}_{o}$, the right-hand-side of (3). Our choice of ${\mathbf {D}}$ is ${\mathbf {I}}d$, where ${\mathbf {I}}$ is an identity matrix and *d* is set to be the largest eigenvalue of ${\mathbf {W}}_{o}^{T}{\mathbf {W}}_{o}$. In practice, a small value, e.g., 0.001, is added to *d* to avoid computationally unstable solutions. A small numerical example of ODA can be found in Appendix.

#### Gibbs sampling for marker effects in the BayesXII algorithm

Employing ODA, Bayesian linear regression models can be written as:

4 $$ \begin{bmatrix}{\mathbf {y}}\\ \widetilde{{\mathbf {y}}} \end{bmatrix}=\begin{bmatrix}{\mathbf {1}}\\ \widetilde{{\mathbf {J}}} \end{bmatrix}\mu +\begin{bmatrix}{\mathbf {X}}\\ \widetilde{{\mathbf {X}}} \end{bmatrix}{\mathbf {a}}+\begin{bmatrix}{\mathbf {e}}\\ \widetilde{{\mathbf {e}}} \end{bmatrix}, $$

where $\widetilde{{\mathbf {y}}}$ denotes a vector of unobserved phenotypes that are introduced into the model, $$\begin{bmatrix}{\mathbf {e}}\\ \widetilde{{\mathbf {e}}} \end{bmatrix}\overset{}{\sim }N\left( {\mathbf {0}},{\mathbf {I}}\sigma _{e}^{2}\right) $$ and $\widetilde{{\mathbf {J}}}$, $\widetilde{{\mathbf {X}}}$ are obtained using (3) with $${\mathbf {W}}_{a}=\begin{bmatrix}\widetilde{{\mathbf {J}}}&\widetilde{{\mathbf {X}}}\end{bmatrix}$$, and $${\mathbf {W}}_{o}=\begin{bmatrix}{\mathbf {1}}&{\mathbf {X}}\end{bmatrix}$$.

In the BayesXII algorithm, the full conditional distribution of $a_{j}$ when $a_{j}$ is non-zero, under model (4), which was derived in Appendix, can be written as:

5 $$ \left( {a_{j} \left| {ELSE} \right.} \right) \sim N\left( {\frac{{{\mathbf {X}}_{j}^{T} \mathbf y + \widetilde{{\mathbf{X}}}_{j}^{T} \widetilde{\mathbf y}}}{{d + \frac{{\sigma _{e}^{2} }}{{\sigma _{a}^{2} }}}},\frac{{\sigma _{e}^{2} }}{{d + \frac{{\sigma _{e}^{2} }}{{\sigma _{a}^{2} }}}}} \right), $$

where the mean and variance parameters are free of the values of the other marker effects $a_{j^{'}\ne j}$. Thus, the full conditional distributions of the marker effects are independent, and thus, samples for each marker can be obtained simultaneously and therefore in parallel. Note that ${\mathbf {X}}_{j}^{T}{\mathbf {y}}$ does not change, and only $\widetilde{{\mathbf {X}}}_{j}^{T}\widetilde{{\mathbf {y}}}$ needs to be computed at each step of the MCMC chain, where the number of operations for this is always of order *p* regardless of the size of *n*. In the BayesXII algorithm, sampling marker effects at each MCMC step, however, requires sampling of the vector $\widetilde{{\mathbf {y}}}$ of unobserved phenotypes. At each step of the MCMC chain, each element of the “missing” phenotypes $\widetilde{{\mathbf {y}}}$ is sampled from independent univariate normal distributions as:

6 $$ \left( {\widetilde{\mathbf y}\left| {ELSE} \right.} \right) \sim N(\widetilde{{\mathbf{J}}}\mu + \widetilde{{\mathbf{X}}}{\text{a, }}{\mathbf{I}}\sigma _{e}^{2} ).  $$

Note that the means of these normal distributions can be computed in parallel as described in Appendix. Once the means are computed, each element in $\widetilde{{\mathbf {y}}}$ can be sampled in parallel. The derivation of the full conditional distributions of other parameters of interest in the BayesXII algorithm and parallel implementation of the BayesXII algorithm using Message Passing Interface (MPI) [15] are shown in Appendix.

## Data analysis

A dataset of 60,000 observations with a medium-density marker panel was simulated using software XSim [16] to compare the BayesXII algorithm with the conventional sampler for BayesC$\pi$. Publicly available genotypes for a medium-density marker panel ($\sim$ 42,000 markers after quality control) were obtained for 100 German Holstein cattle (https://datashare.is.ed.ac.uk/handle/10283/3040). Then, haplotypes of these 100 individuals were estimated from their genotypic data using the software WinHap [17]. Starting from a base population of these 100 individuals, random mating was simulated for 100 generations, and continued for one more generation to increase the population size to 60,000 individuals, which were then used in the analysis. A random sample of five percent of the total number of loci were selected as quantitative trait loci (QTL), and their effects were sampled from a univariate normal distribution with mean zero and variance one. The QTL effects were scaled such that the genetic variance from the last generation was 1.0. A trait with a heritability 0.3 was simulated by adding independent residuals to the genetic values. In our analysis, a randomly sampled subset of 50,000 individuals was used for training and the remaining 10,000 individuals were used for testing.

This dataset was used to compare the BayesXII algorithm with the conventional sampler for BayesC$\pi$. For both BayesXII algorithm and conventional sampler for BayesC$\pi$, five Markov chains of length 100,000 were generated from five different sets of starting values for marker effects sampled from a normal distribution with null mean and variance calculated as $\sigma ^2_a = \frac{\sigma ^2_g}{(1-\pi )\sum 2p_i(1-p_i)}$, where $\sigma ^2_g$ is the genetic variance, $p_i$ is the allele frequency for locus *i*, and $\pi$ is the probability that a marker has a null effect.

Prediction accuracies, which are calculated as the correlation between estimated breeding values and adjusted phenotypic values, in the testing population were used to compare these two methods. The correlation between estimated breeding values of these two methods for the testing population was investigated to: (1) confirm that the BayesXII algorithm can provide about the same prediction accuracy as the conventional sampler for BayesC$\pi$; and (2) quantify the relative convergence of the BayesXII algorithm and conventional sampler for BayesC$\pi$. Our BayesXII algorithm was implemented using the Message Passing Interface (MPI) [15], which is a message-passing standard for distributed memory programming. The speed of our parallel implementation of the BayesXII algorithm was tested on a server with up to 24 nodes in the same rack, and there were 24 cores on each node.

The authors state that all data necessary for confirming the conclusions presented in this article are represented fully within the article.

## Results

The speed of the BayesXII algorithm is shown in Table 1. The total runtime for the BayesXII algorithm was sped up nearly linearly by the number of computer processors. Using 576 processors (24 nodes with 24 cores on each node), the BayesXII algorithm required about 47 min to obtain samples for a chain of length 100,000. However, the conventional sampler for BayesC$\pi$ running on one node and one core required about 7900 minutes for the same chain length, which was about 170 times slower than the BayesXII algorithm.

**Table 1:** Computing time for the BayesXII algorithm to obtain samples for a chain of length 100,000

Number of nodes	Total number of cores	$\text {Runtime}^{\text{a}}$ (min)
2	48	436
5	120	191
10	240	94
15	360	69
20	480	54
24	576	47

$^{\text{a}}$ Note that different number of processes in MPI were tested for different number of nodes (computer used as a server), but only the fastest time is shown

Prediction accuracies were obtained from five chains of length 100,000 for each method. The potential scale reduction factor (PSRF) was used to diagnose the convergence of the Markov chain [18, 19]. Using conventional sampler for BayesC$\pi$, convergence required about 3000 iterations (PSRF value for the marker effect variance was smaller than 1.1), where the prediction accuracy of BayesC$\pi$ was 0.5139. The correlation between estimated breeding values from the BayesXII algorithm with those from the conventional sampler for BayesC$\pi$ for the testing population was larger than 0.99 when the chain for the BayesXII algorithm was longer than 50,500, where the prediction accuracy for the BayesXII algorithm was 0.5104. The trajectory of the prediction accuracy of the BayesXII algorithm is provided in the Appendix. In summary, the BayesXII algorithm requires 17 times more samples than the conventional sampler for BayesC$\pi$ to obtain about the same prediction accuracy. Considering that the BayesXII algorithm was 170 times faster than the conventional sampler for BayesC$\pi$ using 24 nodes with 24 cores on each node (i.e., about 25 minutes for a chain of length 50,500), the BayesXII algorithm required one tenth of the computation time in the conventional sampler for BayesC$\pi$.

## Discussion

Whole-genome Bayesian multiple regression methods are usually computationally intensive, where a MCMC chain comprising tens of thousands of steps is typically used for inference. In this paper, a strategy to parallelize Gibbs sampling for each marker within each step of the MCMC chain was proposed. This parallelization is accomplished using an orthogonal data augmentation strategy, where the marker covariate matrix is augmented with *p* new rows such that its columns are orthogonal. Then, the full conditional distributions of marker effects become independent within each step of the chain, and thus, samples of marker effects within each step can be drawn in parallel. Ideally, the BayesXII algorithm can be accelerated by *k* times, where *k* is the number of computer processors, up to *p* times, where *p* is the number of markers. In this paper, the full conditional distributions that are needed for BayesC$\pi$ with orthogonal data augmentation (BayesXII) were derived and the speed of the BayesXII algorithm was evaluated. In the simulation data with 50,000 individuals and a medium-density marker panel ($\sim$ 50,000 markers), the BayesXII algorithm reached about the same accuracy as the conventional sampler for BayesC$\pi$ in less than 30 minutes on 24 nodes with 24 cores on each node. In this case, the BayesXII algorithm required one tenth of the computation time as conventional sampler for BayesC$\pi$.

### Computation

#### Marker effects

The time and space complexity for sampling marker effects, the most time-consuming task, in the BayesXII algorithm and conventional sampler for BayesC$\pi$, are shown in Table 2, where the big *O* notation [20] is used. Time complexity represents the number of elementary operations, such as multiplication and addition, performed by an algorithm. Space complexity represents the amount of storage required by an algorithm. In Bayesian regression model implementations such as BayesC$\pi$, the most time consuming task is sampling the marker effects from their full conditional distributions. The time complexities for two different computational approaches, BayesC$\pi$-I and BayesC$\pi$-II, for conventional sampler for BayesC$\pi$ are *O*(*npt*) and $O(p^2t)$, for which the details are described in Appendix. In the BayesXII algorithm, however, the marker effects can be sampled in parallel within each step, using (5), and the time complexity is $O(p^2t/k)$. Ideally, the computations at each step of the MCMC chain can be accelerated by *k* times, where *k* is the number of computer processors, up to *p* times, where *p* is the number of markers. In our simulated data, the speed performance of the two computational approaches for conventional sampler for BayesC$\pi$ should be similar and only the first approach is shown in "Results" section.

**Table 2:** Time and space complexity of alternative implementations of Bayesian regression models

Algorithms	Time $\text {complexity}^{\text{a}}$		Space $\text {complexity}^{\text{a}}$
	Marker effects	Missing phenotypes	
BayesC$\pi$-I	$O(npt_1)$	NA	*O*(*np*)
BayesC$\pi$-II	$O(p^2t_1)$	NA	$O(p^2)$
BayesXII	$O(p^2t_2/k)$	$O(p^2t_2/k)$	$O(p^2)$

$^{\text{a}}$Variables include *p*, the number of markers; *n*, the number of observations; *t*1 and *t*2, the number of steps of MCMC required to converge in the BayesXII algorithm and conventional samplers for BayesC$\pi$, respectively; *k*, the number of computer processors

#### Variance components

After marker effects are sampled at each step of the MCMC, the computational complexity for sampling residual variances and marker effects variances in the BayesXII algorithm at each step are *O*(*p*) and *O*(*p*), which are negligible compared with the complexity of sampling marker effects. In conventional samplers for BayesC$\pi$, the computational complexity for sampling residual variances at each step is *O*(*n*) for BayesC$\pi$-I and $O(p^2)$ for BayesC$\pi$-II.

#### Building prerequisite matrices

The time complexities for computations to build prerequisite matrices that are done only once for the BayesC$\pi$-I, BayesC$\pi$-II and BayesXII algorithms on a single-core are *O*(*np*), $O(np^2)$ and $O(np^2+p^3)$. The computing time for these “only-once” computations, however, is trivial compared to that for sampling marker effects. For example, in the BayesXII algorithm, the time complexity for computation to build the augmented matrix ${\mathbf {W}}_{a}$ as in (3) on a single core is $O(np^2+p^3)$. In (3), the two tasks are: (1) computation of ${\mathbf {X}}^{T}{\mathbf {X}}$, where ${\mathbf {X}}$ is an $n\times p$ matrix; and (2) Cholesky decomposition of a positive definite matrix of size *p*. Parallel computing approaches for the first of these two tasks is given in Appendix. The computing time for the Cholesky decomposition in the second task is relatively short, requiring about 5 minutes for $p=50,000$ on a workstation with 4 cores and 64G memory.

### More complicated scenarios

#### Larger sample size

**Fig. 1:**
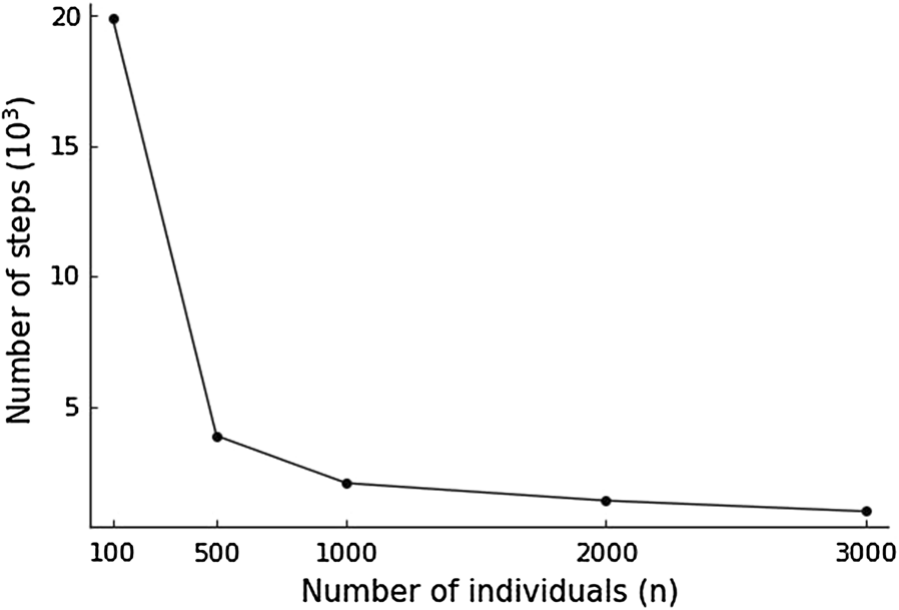
Effect of sample size on the convergence of the BayesXII algorithm. Number of MCMC steps required for the BayesXII algorithm to obtain similar estimated breeding value as conventional sampler for BayesC$\pi$ using a low-density marker panel (1000 markers)

In the BayesXII algorithm, the marker covariate matrix is augmented by adding *p* new rows such that its columns are orthogonal. Compared with the conventional sampler for BayesC$\pi$, the *p* “missing” phenotypes are sampled at each step of the MCMC chain in the BayesXII algorithm. This makes the convergence of the BayesXII algorithm slower than for the conventional sampler. Thus, the ratio of the number *p* of markers and the number *n* of individuals may affect the convergence of the BayesXII algorithm. We simulated new datasets with 1000 markers and different number of individuals. A random sample of five percent of those 1000 markers were selected as QTL. Figure 1 shows the effect of sample size on the convergence of the BayesXII algorithm, where the number of steps required for the BayesXII algorithm to obtain similar estimated breeding value as conventional sampler for BayesC$\pi$ was shown for different *n* with $p=1000$. It can be seen that fewer iterations are needed as more individuals (*n*) become available. This is happening in genomic prediction as more individuals are genotyped.

#### High-density marker panel

In the BayesXII algorithm, the marker covariate matrix is augmented by adding a matrix $\widetilde{{\mathbf {X}}}$ to make all columns of the augmented marker covariate matrix orthogonal to each other. However, with high-density marker panels (e.g., whole-genome sequence data), a large amount of memory will be used to store $\widetilde{{\mathbf {X}}}$, and the Cholesky decomposition of what may be a huge square matrix of size *p* is required, which can be very slow or infeasible. Thus, we propose another approach below. Markers are partitioned into small groups, and a square matrix $\widetilde{{\mathbf {X}}}_i$ is generated to orthogonalize columns of the marker covariate matrix in group *i* such that marker effects for markers in the same group can be sampled in parallel at each step. For example, for a marker covariate matrix partitioned into *m* groups, $${\mathbf {X}}= \begin{bmatrix}{\mathbf {X}}_1&{\mathbf {X}}_2&\cdots&{\mathbf {X}}_m \end{bmatrix}$$, the augmented marker covariate matrix can be written as:

$$ \begin{bmatrix}{\mathbf {X}}\\ \widetilde{{\mathbf {X}}} \end{bmatrix}= \begin{bmatrix}{\mathbf {X}}_1 & {\mathbf {X}}_2 & \cdots & {\mathbf {X}}_m\\ \widetilde{{\mathbf {X}}}_1 & \widetilde{{\mathbf {X}}}_2 & \cdots & \widetilde{{\mathbf {X}}}_m \end{bmatrix}, $$

where

$$ \begin{bmatrix}{\mathbf {X}}_{i}^{T}&\widetilde{{\mathbf {X}}}_{i}^{T} \end{bmatrix}\begin{bmatrix}{\mathbf {X}}_{i} \\ \widetilde{{\mathbf {X}}}_{i} \end{bmatrix}={\mathbf {D}}_{i}, $$

and ${\mathbf {D}}_{i}$ is a diagonal matrix for group *i*. Note that the way in which markers are partitioned into small groups may affect the convergence.

## Conclusions

A fast parallelized algorithm called BayesXII is proposed in this paper. Ideally, the computations at each step of the MCMC chain can be accelerated by *k* times, where *k* is the number of computer processors, up to *p* times, where *p* is the number of markers. In a simulation analysis with 50,000 individuals and a medium-density marker panel, the BayesXII algorithm reached about the same prediction accuracy as the conventional samplers for BayesC$\pi$ within one tenth of the computation time for the conventional sampler. In conclusion, we believe that the BayesXII algorithm is a practical alternative for accelerating Gibbs sampling for some applications of Bayesian regression models, such as those encountered in genomic prediction. Addressing the heavy computational burden associated with Bayesian regression models by parallel computing will lead to greater use of these models.

## Figures and Tables

**Fig. 2:**
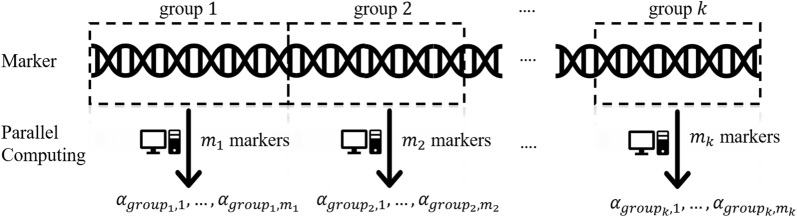
Parallel implementation for sampling marker effects at each step in the BayesXII algorithm. The *i*-th process will calculate the number of $m_i$ marker effects on group *i*, and $\sum _{i=1}^{k} m_i=p$

**Fig. 3:**
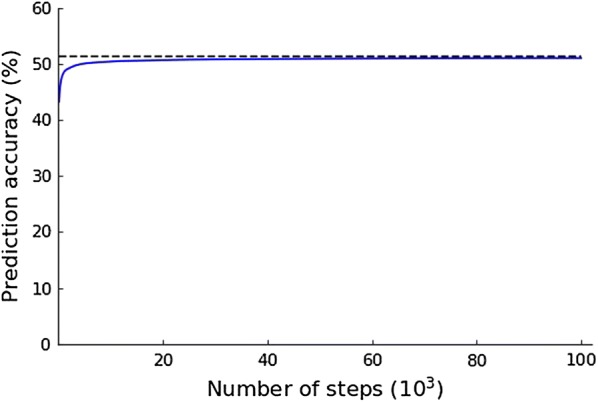
Prediction accuracy of the BayesXII algorithm. The blue solid line is the prediction accuracy of the BayesXII algorithm for BayesC$\pi$. The black dashed line is the prediction accuracy obtained from the converged conventional sampler for BayesC$\pi$

## Appendix

### Two computational approaches for the conventional sampler for BayesC$\pi$

Detailed derivation of the full conditional distributions of the marker effect for locus *j* in conventional sampler for BayesC$\pi$ is in Fernando and Garrick [21]. As shown in [21], the full conditional distribution of $a_{j}$ in BayesC$\pi$, when $a_{j}$ is non-zero, is

7 $$ \left( {a_{j} \left| {ELSE} \right.} \right) \sim N\left( {\hat{a}_{j} ,\frac{{\sigma _{e}^{2} }}{{{\mathbf{X}}_{j}^{T} {\mathbf{X}}_{{\text{j}}}  + \frac{{\sigma _{e}^{2} }}{{\sigma _{a}^{2} }}}}} \right) $$

where *ELSE* stands for all the other unknowns and ${\mathbf {y}}$, ${\mathbf {X}}_{j}$ is the *j*th column of ${\mathbf {X}}$, and $\hat{a_{j}}$ is the solution to:

8 $$ \left( {{\bf{X}}_{j}^{T} {\bf{X}}_{j}  + \frac{{\sigma _{e}^{2} }}{{\sigma _{a}^{2} }}} \right)\hat{a}_{j}  = {{\bf X}}_{j}^{T} \left( {{\bf y} - {\mathbf{1}}\mu  - \sum\limits_{{j^{\prime} \ne j}} {{\bf X}_{{j^{\prime}}} a_{j^{\prime}} } } \right) $$

9 $$= {\mathbf {X}}_{j}^{T}{\mathbf {y}}-{\mathbf {X}}_{j}^{T}{\mathbf {1}}\mu -\sum _{j^{\prime}\ne j}{\mathbf {X}}_{j}^{T}{\mathbf {X}}_{j^{\prime}}a_{j^{\prime}}. $$

It is worth noting that two approaches are available to compute the right-hand-side of (8). The first approach, BayesC$\pi$-I, corresponding to (8) is briefly described below.

1. Initially:
  - Compute $${\mathbf {X}}_{j}^{T}{\mathbf {X}}_{j}$$ with $$j=1,2,3,\ldots ,p,$$
  - Compute ${\mathbf {y}}_{corr}={\mathbf {y}}-{\mathbf {1}}\mu -\sum {\mathbf {X}}_{j} a_{j}$, which is the vector ${\mathbf {y}}$ corrected for all fixed and random effects, using their current values, and
  - Store ${\mathbf {X}}$ in the memory.
2. Then, for each locus at each step of MCMC:
  - Before sampling the marker effect, the right-hand-side of (8) is updated as
  $$ {\mathbf {X}}_{j}^{T}\left( {\mathbf {y}}-{\mathbf {1}}\mu -\sum _{j^{\prime}\ne j}{\mathbf {X}}_{j^{\prime}}a_{j^{\prime}}\right) ={\mathbf {X}}_{j}^{T}{\mathbf {y}}_{corr}+{\mathbf {X}}_{j}^{T}{\mathbf {X}}_{j}a_{j}, $$
  then
  - once a new value of $a_j$, denoted by $a_j^{*}$, is sampled, ${\mathbf {y}}_{corr}$ is updated as
    $$ {\mathbf {y}} _{corr}= {\mathbf {y}}_{corr}+{\mathbf {X}}_{j}^{T}\left( a_j-a_j^{*}\right) . $$

The second approach, BayesC$\pi$-II, corresponding to (9) is briefly described below.

1. Initially:
  - Compute ${\mathbf {X}}^{T}{\mathbf {X}}$, ${\mathbf {X}}^{T}{\mathbf {1}}$ and ${\mathbf {X}}^{T}{\mathbf {y}}$, then,
  - Store these matrices in memory.
2. Then for each locus at each step of MCMC:
  - Before sampling the marker effect, the right-hand-side is computed as
  $$ \left( {\mathbf {X}}^{T}{\mathbf {y}}\right) _{j}-\left( {\mathbf {X}}^{T}{\mathbf {1}}\right) _{j}\mu -\left( {\mathbf {X}}^{T}{\mathbf {X}}\right) _{:,j}{\mathbf {a}}+\left( {\mathbf {X}}^{T}{\mathbf {X}}\right) _{j,j}a_{j}, $$

where $\left( {\mathbf {X}}^{T}{\mathbf {X}}\right) _{:,j}$ is the *j*th column of ${\mathbf {X}}^{T}{\mathbf {X}}$ and $\left( {\mathbf {X}}^{T}{\mathbf {X}}\right) _{j,j}$ is the jth diagonal element of ${\mathbf {X}}^{T}{\mathbf {X}}$. Note that the number of operations to sample marker effects for each locus at each step of the MCMC is of order *n* in the first approach and of order *p* for the second approach. Thus, the computational complexities for sampling marker effects in conventional sampler for BayesC$\pi$ are *O*(*npt*) for the first approach and $O(p^2t)$ for the the second approach. The space complexities are *O*(*np*) for storing ${\mathbf {X}}$ in the first approach and $O(p^2)$ for storing ${\mathbf {X}}^{T}{\mathbf {X}}$ in the second approach.

### Single-site Gibbs sampler for the BayesXII algorithm

#### Full conditional distribution of the marker effect

The full conditional distribution of $a_{j}$ in the BayesXII algorithm, which is shown below, can be obtained from (7) and (8) by replacing $\mathbf {{y}}$ with $$\begin{bmatrix}{\mathbf {y}}\\ \widetilde{{\mathbf {y}}} \end{bmatrix}$$, $\mathbf {{1}}$ with $$\begin{bmatrix}{\mathbf {1}}\\ \widetilde{{\mathbf {J}}} \end{bmatrix}$$ and $\mathbf {{X}}$ with $$\begin{bmatrix}{\mathbf {X}}\\ \widetilde{{\mathbf {X}}} \end{bmatrix}$$. Note that columns of the augmented covariate matrix $$\begin{bmatrix}{\mathbf {1}} &{} {\mathbf {X}}\\ \widetilde{{\mathbf {J}}} &{} \widetilde{{\mathbf {X}}} \end{bmatrix}$$ are orthogonal. Thus, (7) for the BayesXII algorithm can be simplified as:

10 $$\begin{aligned} \left( \begin{bmatrix}{\mathbf {X}}_{j}^{T}\widetilde{{\mathbf {X}}}_{j}^{T}\end{bmatrix}\begin{bmatrix}{\mathbf {X}}_{j}\\ \widetilde{{\mathbf {X}}}_{j} \end{bmatrix}+\frac{\sigma _{e}^{2}}{\sigma _{a}^{2}}\right) \hat{a_{j}}= & {} \begin{bmatrix}{\mathbf {X}}_{j}^{T}\widetilde{{\mathbf {X}}}_{j}^{T}\end{bmatrix}\left( \mathbf {\begin{bmatrix}{\mathbf {y}}\\ \widetilde{{\mathbf {y}}} \end{bmatrix}}-\begin{bmatrix}{\mathbf {1}}\\ \widetilde{{\mathbf {J}}} \end{bmatrix}\mu - \sum _{j^{\prime}\ne j}\begin{bmatrix}{\mathbf {X}}_{j^{\prime}}\\ \widetilde{{\mathbf {X}}}_{j^{\prime}} \end{bmatrix}a_{j^{\prime}}\right) \nonumber \\ \left( \begin{bmatrix}{\mathbf {X}}_{j}^{T}\widetilde{{\mathbf {X}}}_{j}^{T}\end{bmatrix}\begin{bmatrix}{\mathbf {X}}_{j}\\ \widetilde{{\mathbf {X}}}_{j} \end{bmatrix}+\frac{\sigma _{e}^{2}}{\sigma _{a}^{2}}\right) \hat{a_{j}}= & {} \begin{bmatrix}{\mathbf {X}}_{j}^{T}\widetilde{{\mathbf {X}}}_{j}^{T}\end{bmatrix}\mathbf {\begin{bmatrix}{\mathbf {y}}\\ \widetilde{{\mathbf {y}}} \end{bmatrix}}-\begin{bmatrix}{\mathbf {X}}_{j}^{T}\widetilde{{\mathbf {X}}}_{j}^{T}\end{bmatrix}\begin{bmatrix}{\mathbf {1}}\\ \widetilde{{\mathbf {J}}} \end{bmatrix}\mu - \nonumber \\&\sum _{j^{\prime}\ne j}\begin{bmatrix}{\mathbf {X}}_{j}^{T}\widetilde{{\mathbf {X}}}_{j}^{T}\end{bmatrix}\begin{bmatrix}{\mathbf {X}}_{j^{\prime}}\\ \widetilde{{\mathbf {X}}}_{j^{\prime}} \end{bmatrix}a_{j^{\prime}}\nonumber \\ \left( d+\frac{\sigma _{e}^{2}}{\sigma _{a}^{2}}\right) \hat{a_{j}}= & {} \begin{bmatrix}{\mathbf {X}}_{j}^{T}\widetilde{{\mathbf {X}}}_{j}^{T}\end{bmatrix}\mathbf {\begin{bmatrix}{\mathbf {y}}\\ \widetilde{{\mathbf {y}}} \end{bmatrix}}\nonumber \\ \hat{a_{j}}= & {} \frac{{\mathbf {X}}_{j}^{T}{\mathbf {y}}+\widetilde{{\mathbf {X}}}_{j}^{T}\widetilde{{\mathbf {y}}}}{d+\frac{\sigma _{e}^{2}}{\sigma _{a}^{2}}}. \end{aligned}$$

Thus, the full conditional distribution of $a_{j}$ can be written as:

$$ \left( a_{j}\mid ELSE\right)  \sim N\left( \frac{{\mathbf {X}}_{j}^{T}{\mathbf {y}}+\widetilde{{\mathbf {X}}}_{j}^{T}\widetilde{{\mathbf {y}}}}{d+\frac{\sigma _{e}^{2}}{\sigma _{a}^{2}}},\frac{\sigma _{e}^{2}}{d+\frac{\sigma _{e}^{2}}{\sigma _{a}^{2}}}\right) . $$

Note that the number of operations is of order *p* for sampling marker effects, thus the computational complexity is $O(p^2t)$. The space complexity is $O(p^2)$ for $\widetilde{{\mathbf {X}}}$.

Detailed derivation of the full conditional distribution of the indicator variable $\delta _{j}$ indicating if $a_{j}$ had a normal distribution ($\delta _{j}=1$) or if it is null ($\delta _{j}=0$) in the conventional sampler for BayesC$\pi$ is also in Fernando and Garrick [21]. The full conditional distribution of $\delta _{j}$ in conventional sampler for BayesC$\pi$ is:

11 $$\begin{aligned}&Pr\left( \delta _{j}=1\mid ELSE\right) \nonumber \\&\quad =\frac{f_{1}\left( r_{j}\mid \sigma _{a}^{2},\sigma _{e}^{2}\right) Pr\left( \delta _{j}=1\right) }{f_{0}\left( r_{j}\mid \sigma _{e}^{2}\right) Pr\left( \delta _{j}=0\right) +f_{1}\left( r_{j}\mid \sigma _{a}^{2},\sigma _{e}^{2}\right) Pr\left( \delta _{j}=1\right) }, \end{aligned}$$

where $f_{1}\left( r_{j}\mid \sigma _{a}^{2},\sigma _{e}^{2}\right)$ is a univariate normal distribution with

$$ E\left( r_{j}\mid \sigma _{a}^{2},\sigma _{e}^{2}\right) =0,Var\left( r_{j}\mid \sigma _{a}^{2},\sigma _{e}^{2}\right) =\left( {\mathbf {X}}_{j}^{T}{\mathbf {X}}_{j}\right) ^{2}\sigma _{a}^{2}+{\mathbf {X}}_{j}^{T}{\mathbf {X}}_{j}\sigma _{e}^{2} , $$

and $f_{0}\left( r_{j}\mid \sigma _{e}^{2}\right)$ is a univariate normal distribution with

$$ E\left( r_{j}\mid \sigma _{e}^{2}\right) =0, Var\left( r_{j}\mid \sigma _{e}^{2}\right) ={\mathbf {X}}_{j}^{T}{\mathbf {X}}_{j}\sigma _{e}^{2}, $$

and

$$ r_{j}={\mathbf {X}}_{j}^{T}\left( {\mathbf {y}}-{\mathbf {1}}\mu -\sum _{j^{\prime}\ne j}{\mathbf {X}}_{j^{\prime}}a_{j^{\prime}}\right) .$$

The full conditional distribution of $\delta _{j}$ in the BayesXII algorithm, which is shown below, can be obtained from (11) by replacing $\mathbf {{y}}$ with $$\begin{bmatrix}{\mathbf {y}}\\ \widetilde{{\mathbf {y}}} \end{bmatrix}$$, $\mathbf {{1}}$ with $$\begin{bmatrix}{\mathbf {1}}\\ \widetilde{{\mathbf {J}}} \end{bmatrix}$$ and $\mathbf {{X}}$ with $$\begin{bmatrix}{\mathbf {X}}\\ \widetilde{{\mathbf {X}}} \end{bmatrix}$$. Thus, (11) for the BayesXII algorithm can be simplified as:

$$\begin{aligned}&Pr\left( \delta _{j}=1\mid ELSE\right) \\&\quad =\frac{f_{1}\left( r_{j}\mid \sigma _{a}^{2},\sigma _{e}^{2}\right) Pr\left( \delta _{j}=1\right) }{f_{0}\left( r_{j}\mid \sigma _{e}^{2}\right) Pr\left( \delta _{j}=0\right) +f_{1}\left( r_{j}\mid \sigma _{a}^{2},\sigma _{e}^{2}\right) Pr\left( \delta _{j}=1\right) }, \end{aligned}$$

where $f_{1}\left( r_{j}\mid \sigma _{a}^{2},\sigma _{e}^{2}\right)$ is a univariate normal with

$$\begin{aligned} E\left( r_{j}\mid \sigma _{a}^{2},\sigma _{e}^{2}\right) =0,&Var\left( r_{j}\mid \sigma _{a}^{2},\sigma _{e}^{2}\right) =d^{2}\sigma _{a}^{2}+d\sigma _{e}^{2}, \end{aligned}$$

and $f_{0}\left( r_{j}\mid \sigma _{e}^{2}\right)$ is a univariate normal with

$$\begin{aligned} E\left( r_{j}\mid \sigma _{e}^{2}\right) =0,&Var\left( r_{j}\mid \sigma _{e}^{2}\right) =d\sigma _{e}^{2}, \end{aligned}$$

and

$$\begin{aligned} r_{j}={\mathbf {X}}_{j}^{T}{\mathbf {y}}+\widetilde{{\mathbf {X}}}_{j}^{T}\widetilde{{\mathbf {y}}}. \end{aligned}$$

#### Full conditional distributions of the unobserved phenotypes

The full conditional distribution of $\widetilde{{\mathbf {y}}}$ can be written as:

$$\begin{aligned} f\left( \widetilde{{\mathbf {y}}}\mid {\mathbf {a}},\mu ,\sigma _{e}^{2},\sigma _{a}^{2},{\mathbf {y}}\right)&=f\left( \widetilde{{\mathbf {y}}}\mid {\mathbf {a}},\mu ,\sigma _{e}^{2}\right) \\&\propto N\left( \widetilde{{\mathbf {J}}}\mu +\widetilde{{\mathbf {X}}}{\mathbf {a}},{\mathbf {I}}\sigma _{e}^{2}\right) . \end{aligned}$$

#### Full conditional distributions of other unknowns

The derivation of the full conditional distributions of other parameters such as $\mu$, $\sigma _{a}^{2}$, $\sigma _{e}^{2}$, $\pi$ are straightforward. Thus they are presented as below without derivations.

$$\begin{aligned} \left( \mu \mid ELSE\right)&\sim N\left( \frac{{\mathbf {1}}^{T}{\mathbf {y}}+\widetilde{{\mathbf {J}}}^{T}\widetilde{{\mathbf {y}}}}{d},\frac{\sigma _{e}^{2}}{d}\right) ,\\ \left( \sigma _{a}^{2}\mid ELSE\right)&\sim \left( {\mathbf {a}}^{T}{\mathbf {a}}+\nu _{a}S_{a}^{2}\right) \chi _{k+\nu _{a}}^{-2},\\ \left( \sigma _{e}^{2}\mid ELSE\right)&\sim \left( {\mathbf {y}}_{corr}^{T}{\mathbf {y}}_{corr}+\nu _{e}S_{e}^{2}\right) \chi _{n+p+\nu _{e}}^{-2},\\ \left( \pi \mid ELSE\right)&\sim Beta(p - k + 1,k + 1), \end{aligned}$$

where $${\mathbf {y}}_{corr}=\mathbf {\begin{bmatrix}{\mathbf {y}}\\ \widetilde{{\mathbf {y}}} \end{bmatrix}}-\begin{bmatrix}{\mathbf {1}}\\ \widetilde{{\mathbf {J}}} \end{bmatrix}\mu -\begin{bmatrix}{\mathbf {X}}\\ \widetilde{{\mathbf {X}}} \end{bmatrix}{\mathbf {a}}$$, *k* is the number of markers in the model at each step. Note that ${\mathbf {y}}_{corr}^{T}{\mathbf {y}}_{corr}$ can be written as:

$$\begin{aligned}&\left( \mathbf {\begin{bmatrix}{\mathbf {y}}\\ \widetilde{{\mathbf {y}}} \end{bmatrix}}-\begin{bmatrix}{\mathbf {1}}\\ \widetilde{{\mathbf {J}}} \end{bmatrix}\mu -\begin{bmatrix}{\mathbf {X}}\\ \widetilde{{\mathbf {X}}} \end{bmatrix}{\mathbf {a}}\right) ^{T}\left( \mathbf {\begin{bmatrix}{\mathbf {y}}\\ \widetilde{{\mathbf {y}}} \end{bmatrix}}-\begin{bmatrix}{\mathbf {1}}\\ \widetilde{{\mathbf {J}}} \end{bmatrix}\mu -\begin{bmatrix}{\mathbf {X}}\\ \widetilde{{\mathbf {X}}} \end{bmatrix}{\mathbf {a}}\right) \\ & ={\mathbf {y}}^{T}{\mathbf {y}}+\widetilde{{\mathbf {y}}}^{T}\widetilde{{\mathbf {y}}}+d\mu ^{2}+d{\mathbf {a}}^{T}{\mathbf {a}} -2\mu \left( {\mathbf {1}}^{T}{\mathbf {y}}+\widetilde{{\mathbf {J}}}^{T}\widetilde{{\mathbf {y}}}\right) -2{\mathbf {a}}^{T}\left( {\mathbf {X}}^{T}{\mathbf {y}}+\widetilde{{\mathbf {X}}}^{T}\widetilde{{\mathbf {y}}}\right) . \end{aligned}$$

### Parallel implementation of the BayesXII algorithm

As shown above, in the BayesXII algorithm, all marker effects can be sampled simultaneously in parallel within each step of the chain. Given *k* available computer processes, markers can be split into *k* groups, and each computer process can be used to sample marker effects in the corresponding group. A graph to demonstrate such a parallel implementation for sampling marker effects at each step is shown in Fig. 2.

#### Parallel computing of $\widetilde{{\mathbf {X}}}{\mathbf {a}}$

In many modern programming languages, such as R, Python and Julia, libraries are available to take advantage of multiple processors and GPUs for parallel computing of many matrix or vector operations. The descriptions given below are only to illustrate the main principle underlying parallel computing, which involves distributing calculations across processors. Actual implementations may be different and will depend on the programming language, the library and the hardware used.

To sample the unobserved phenotypic values using (6), a matrix by vector product $\widetilde{{\mathbf {X}}}{\mathbf {a}}$ is needed. Here we describe how parallel computing can be used to compute the product of a matrix $\widetilde{{\mathbf {X}}}$ by a vector ${\mathbf {a}}$.

1. Partition $\widetilde{{\mathbf {X}}}$ of size $n\times p$ by columns into smaller submatrices $\widetilde{{\mathbf {X}}}_{(1)},\widetilde{{\mathbf {X}}}_{(2)},\widetilde{{\mathbf {X}}} _{(3)},\ldots$of size $n\times p_{i}$, and partition ${\mathbf {a}}$ into smaller subvectors ${\mathbf {a}}_{(1)},{\mathbf {a}}_{(2)},{\mathbf {a}}_{(3)},\ldots$ of length $p_{i}$ with $\sum p_{i}=p$.
2. Compute $\widetilde{{\mathbf {X}}}{\mathbf {a}}$ as $\widetilde{{\mathbf {X}}}_{(1)}{\mathbf {a}}_{(1)}+\widetilde{{\mathbf {X}}}_{(2)}{\mathbf {a}}_{(2)}+\widetilde{{\mathbf {X}}}_{(3)}{\mathbf {a}}_{(3)}+\ldots$, where $\widetilde{{\mathbf {X}}}_{(i)}{\mathbf {a}}_{(i)}$ for $i=1,2,\ldots$ are computed on different processors and then summed to obtain $\widetilde{{\mathbf {X}}}{\mathbf {a}}$.

Note that the same strategy can also be used to calculate ${\mathbf {X}}^{T}{\mathbf {y}}$ by partitioning ${\mathbf {X}}$ by rows.

#### Parallel computing of ${\mathbf {X}}^{T}{\mathbf {X}}$

In (3), computation of ${\mathbf {X}}^{T}{\mathbf {X}}$ is needed. Here we describe how parallel computing can be used to compute ${\mathbf {X}}^{T}{\mathbf {X}}$, where ${\mathbf {X}}$ is a $n\times p$ matrix.

1. Partition ${\mathbf {X}}$ of size $n\times p$ by rows into smaller submatrices ${\mathbf {X}}_{(1)},{\mathbf {X}}_{(2)},{\mathbf {X}}_{(3)},\ldots$of size $n_{i}\times p$ with $\sum n_{i}=n$.
2. Compute ${\mathbf {X}}^{T}{\mathbf {X}}={\mathbf {X}}_{(1)}^{T}{\mathbf {X}}_{(1)} + {\mathbf {X}}_{(2)}^{T}{\mathbf {X}}_{(2)}+\ldots$, where ${\mathbf {X}}_{(j)}^{T}{\mathbf {X}}_{(j)}$ for $j=1,2,\ldots$ are computed on different processors and then summed to obtain ${\mathbf {X}}^{T}{\mathbf {X}}$.

Note that once ${\mathbf {X}}^{T}{\mathbf {X}}$ has been built, it can be updated as ${\mathbf {X}}^{T}{\mathbf {X}}+{\mathbf {X}}_{new}^{T}{\mathbf {X}}_{new}$ when new observations ${\mathbf {X}}_{new}$ of size $n_{new}\times p$ are available, for which the computation complexity is $O(p^2n_{new})$. The computing time is trivial since $n_{new}$ is usually small. In addition to the benefit of reducing the computing time, this approach can also address the limitation that ${\mathbf {X}}$ may be too large to be stored on a single computing node ($n\gg p$) by distributing the ${\mathbf {X}}_{(i)}$ across several nodes.

### A numerical example of ODA

A small numerical example of ODA is provided here to illustrate the orthogonal data augmentation (ODA). Assuming there are only three individuals and five markers, the centered $3 \times 5$ marker covariate matrix is:

$$\begin{aligned} {\mathbf {X}}=\begin{bmatrix} -0.33 &{}-0.33 &{} 0.67&{} -0.33&{} -1.33\\ 0.67 &{}-0.33 &{} 0.67 &{} 0.67&{} 0.67\\ -0.33 &{}0.67 &{} -1.33 &{}-0.33&{} 0.67 \end{bmatrix}. \end{aligned}$$

Then, the ${\mathbf {W}}_{o}$ is

$$\begin{aligned} {\mathbf {W}}_{o}=\begin{bmatrix}{\mathbf {1}}&{\mathbf {X}}\end{bmatrix}= \begin{bmatrix} 1 &{} -0.33 &{}-0.33 &{} 0.67&{} -0.33&{} -1.33\\ 1 &{} 0.67 &{}-0.33 &{} 0.67 &{} 0.67&{} 0.67\\ 1 &{} -0.33 &{}0.67 &{} -1.33 &{}-0.33&{} 0.67 \end{bmatrix}. \end{aligned}$$

The *d* is set to be the largest eigenvalue of ${\mathbf {W}}_{o}^{T}{\mathbf {W}}_{o}$, which is about 4.55 in this example.

In (3), ${\mathbf {W}}_{a}^{T}{\mathbf {W}}_{a}$ can be calculated as:

$$\begin{aligned} {\mathbf {W}}_{a}^{T}{\mathbf {W}}_{a}&={\mathbf {D}}-{\mathbf {W}}_{o}^{T}{\mathbf {W}}_{o} \\ & ={\mathbf {I}}d-{\mathbf {W}}_{o}^{T}{\mathbf {W}}_{o} \\ & =\begin{bmatrix} 1.55 & 0.0 & 0.0 & 0.0 & 0.0 & 0.0 \\ 0.0 & 3.88 & 0.33 & -0.67 & -0.67 & -0.67\\ 0.0 & 0.33 & 3.88 & 1.33 & 0.33 & -0.67\\ 0.0 & -0.67 & 1.33 & 1.88 & -0.67 & 1.33\\ 0.0 & -0.67 & 0.33 & -0.67 & 3.88 & -0.67\\ 0.0 & -0.67 & -0.67 & 1.33 & -0.67 & 1.88 \end{bmatrix}. \end{aligned}$$

In this example, ${\mathbf {W}}_a$ is obtained using Cholesky decomposition of ${\mathbf {W}}_{a}^{T}{\mathbf {W}}_{a}$, and ${\mathbf {W}}_{a}$ is

$$\begin{aligned} {\mathbf {W}}_{a} = \begin{bmatrix} 1.24 &{} 0.0 &{} 0.0 &{} 0.0 &{} 0.0 &{} 0.0 \\ 0.0 &{} 1.97 &{} 0.17 &{} -0.34 &{} -0.34&{} -0.34\\ 0.0 &{} 0.0 &{} 1.96 &{} 0.71 &{} 0.2 &{} -0.31\\ 0.0 &{} 0.0 &{} 0.0 &{} 1.13 &{} -0.82 &{} 1.28\\ 0.0 &{} 0.0 &{} 0.0 &{} 0.0 &{} 1.75 &{} 0.19\\ 0.0 &{} 0.0 &{} 0.0 &{} 0.0 &{} 0.0 &{} 0.05 \end{bmatrix}. \end{aligned}$$

$\widetilde{{\mathbf {J}}}$ equals the first column of ${\mathbf {W}}_{a}$, and $\widetilde{{\mathbf {X}}}$ is the rest of columns of ${\mathbf {W}}_{a}$ since $${\mathbf {W}}_{a}=\begin{bmatrix}\widetilde{{\mathbf {J}}}&\widetilde{{\mathbf {X}}}\end{bmatrix}$$.

### Prediction accuracy of the BayesXII algorithm

Prediction accuracies of the BayesXII algorithm for BayesC$\pi$ were obtained from five chains of length 100,000. In Fig. 3, the blue solid line is the average of prediction accuracies from those five chains, and the black dashed line is the prediction accuracy obtained from the converged conventional sampler. In summary, the BayesXII algorithm could provide about the same prediction accuracy as the conventional sampler for BayesC$\pi$ when the chain was of length 50,500.
